# *N*-(3,4-Dimethyl­phen­yl)-4-hydr­oxy-2-methyl-2*H*-1,2-benzothia­zine-3-carboxamide 1,1-dioxide

**DOI:** 10.1107/S1600536809010058

**Published:** 2009-03-28

**Authors:** Waseeq Ahmad Siddiqui, Muhammad Ali, Muhammad Zia-ur-Rehman, Saima Sharif, Graham John Tizzard

**Affiliations:** a Department of Chemistry, University of Sargodha, Sargodha, Pakistan; b Applied Chemistry Research Centre, PCSIR Laboratories Complex, Ferozpure Road, Lahore 54600, Pakistan; c Division of Science and Technology, University of Education, Township Campus, College Road, Township, Lahore 54770, Pakistan; d School of Chemistry, University of Southampton, England

## Abstract

1,2-Benzothia­zines similar to the title compound, C_18_H_18_N_2_O_4_S, are well known in the literature for their biological activities and are used as medicines in the treatment of inflammation and rheumatoid arthritis. The thia­zine ring adopts a distorted half-chair conformation. The enolic H atom is involved in an intra­molecular O—H⋯O hydrogen bond, forming a six-membered ring. In the crystal, mol­ecules arrange themselves into centrosymmetric dimers by means of pairs of weak inter­molecular N—H⋯O hydrogen bonds.

## Related literature

For the synthesis of related mol­ecules, see: Siddiqui *et al.* (2007 ▶); Zia-ur-Rehman *et al.* (2005 ▶). For the biological activity of 1,2-benzothia­zine-1,1-dioxides, see: Turck *et al.* (1996 ▶); Zia-ur-Rehman *et al.* (2006 ▶, 2009 ▶). For related structures, see: Golič & Leban (1987 ▶). For the pharmacological background to 1,2-benzothia­zine-3-carboxamide 1,1-dioxide derivatives, see Gennari *et al.* (1994 ▶); Bihovsky *et al.* (2004 ▶).
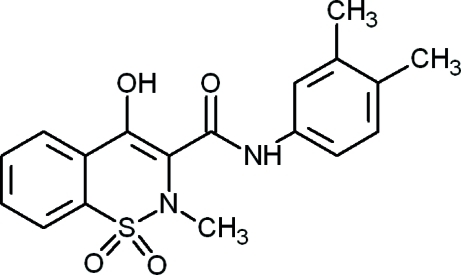


## Experimental

### 

#### Crystal data

C_18_H_18_N_2_O_4_S
*M* *_r_* = 358.40
Triclinic, 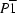

*a* = 7.5458 (4) Å
*b* = 8.0214 (3) Å
*c* = 14.4832 (7) Å
α = 89.864 (3)°
β = 79.530 (2)°
γ = 73.812 (3)°
*V* = 826.78 (7) Å^3^
*Z* = 2
Mo *K*α radiation
μ = 0.22 mm^−1^
*T* = 120 K
0.27 × 0.13 × 0.03 mm

#### Data collection

Bruker–Nonius CCD camera on κ-goniostat diffractometer
Absorption correction: multi-scan (*SADABS*; Sheldrick, 2007 ▶) *T*_min_ = 0.942, *T*_max_ = 0.993
13798 measured reflections
3783 independent reflections
2808 reflections with *I* > 2σ(*I*)
*R*_int_ = 0.055

#### Refinement

*R*[*F* ^2^ > 2σ(*F* ^2^)] = 0.048
*wR*(*F* ^2^) = 0.119
*S* = 1.05
3783 reflections
230 parameters
H-atom parameters constrained
Δρ_max_ = 0.27 e Å^−3^
Δρ_min_ = −0.45 e Å^−3^

### 

Data collection: *COLLECT* (Hooft, 1998 ▶); cell refinement: *DENZO* (Otwinowski & Minor, 1997 ▶) and *COLLECT*; data reduction: *DENZO* and *COLLECT*; program(s) used to solve structure: *SHELXS97* (Sheldrick, 2008 ▶); program(s) used to refine structure: *SHELXL97* (Sheldrick, 2008 ▶); molecular graphics: *SHELXTL* (Sheldrick, 2008 ▶); software used to prepare material for publication: *SHELXTL* and local programs.

## Supplementary Material

Crystal structure: contains datablocks I, global. DOI: 10.1107/S1600536809010058/bt2906sup1.cif

Structure factors: contains datablocks I. DOI: 10.1107/S1600536809010058/bt2906Isup2.hkl

Additional supplementary materials:  crystallographic information; 3D view; checkCIF report

## Figures and Tables

**Table 1:** Hydrogen-bond geometry (Å, °)

*D*—H⋯*A*	*D*—H	H⋯*A*	*D*⋯*A*	*D*—H⋯*A*
O4—H4⋯O3	0.84	1.80	2.545 (2)	146
N2—H2⋯O1^i^	0.88	2.39	3.231 (2)	161

Symmetry code: (i) 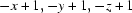.

**Fig. 1.**
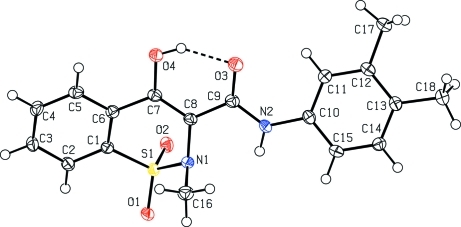
The molecular structure of (I), with displacement ellipsoids at the 50% probability level.

**Fig. 2.**
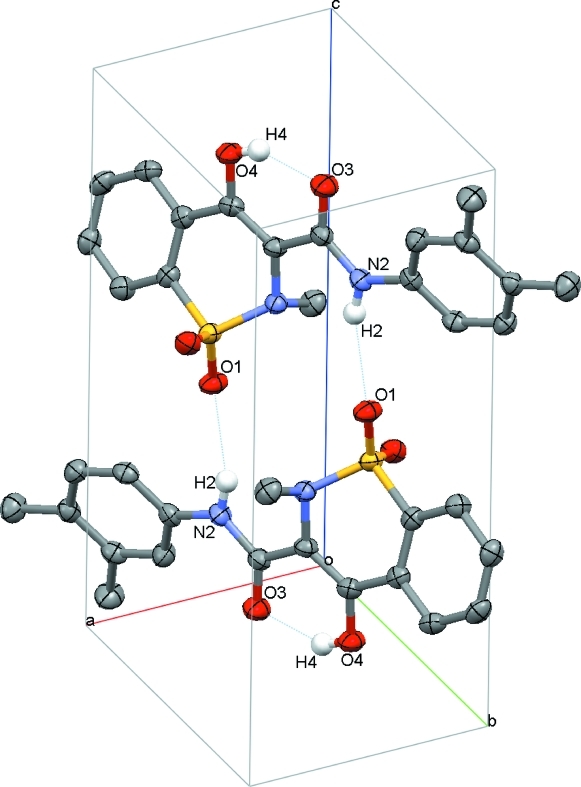
Perspective view of the three-dimensional crystal packing showing hydrogen-bonded interactions (dashed lines). H atoms not involved in hydrogen bonding have been omitted for clarity.

## supplementary crystallographic information

### Comment

In order to discover new useful therapeutic agents, many new compounds are continuously being synthesized. Owing to their applications as non-steroidal anti-inflammatory compounds (Turck *et al.*, 1996), considerable attention has been given to 1,2-benzothiazine 1,1-dioxides and their precursor intermediates (Golič & Leban, 1987). Some of the 1,2-benzothiazines are also known as potent calpain I inhibitors (Bihovsky *et al.*, 2004), while benzothiaine-3-yl-quinazolin-4-ones showed marked activity against Bacillus *subtilis *(Zia-ur-Rehman *et al.*, 2006). 1,2-Benzothiazines are also found to be used for the treatment of rheumatoid arthritis, ankylosing spondylitis, osteoarthrosis and other inflammatory rheumatic and non- rheumatic processes, including onsets and traumatologic lesions (Gennari *et al., *1994). As part of a research program synthesizing various bioactive benzothiazines (Siddiqui *et al.*, 2007, Zia-ur-Rehman *et al.*, 2005, 2006, 2009), we herein report the crystal structure of the title compound  (Scheme and figure 1). The thiazine ring, involving two double bonds, exhibits a distorted half-chair conformation. The enolic hydrogen on O1 is involved in intramolecular hydrogen bonding giving rise to a six-membered hydrogen bond ring (Table 1). The molecules form centrosymmetric dimers through intermolecular N—H···O hydrogen bonds.

### Experimental

A mixture of methyl 4-hydroxy-2-methyl-2*H*-1,2-benzothiazine-3-carboxylate-1,1-dioxide (2.693 g; 10.0 mmoles), 3,4-dimethyl aniline (1.818 g; 15.0 mmoles) and xylene (25.0 ml) was refluxed under nitrogen atmosphere in a Soxhlet apparatus having Linde type 4Å molecular sieves. Three fourth of the xylene was then distilled off and the remaining contents were allowed to stand overnight at room temperature. Settled solids were filtered off, washed with diethyl ether and crystallized from ethanol. Yield: 79.5%.

### Refinement

All hydrogen atoms were identified in the difference map and subsequently fixed in ideal positions and treated as riding on their parent atoms. In the case of the methyl and hydroxyl H atoms the torsion angles were   refined. The following distances were used: C_methyl_—H 0.98 Å; C_aromatic_—H 0.95 Å;  O—H 0.84 Å. U(H) was set to 1.2Ueq of the parent atoms or 1.5Ueq for methyl groups.

### Crystal data

**Table:**

C_18_H_18_N_2_O_4_S	*Z* = 2
*M**_r_* = 358.40	*F*(000) = 376
Triclinic, *P*1	*D*_x_ = 1.440 Mg m^−^^3^
Hall symbol: -P 1	Mo *K*α radiation, λ = 0.71073 Å
*a* = 7.5458 (4) Å	Cell parameters from 8399 reflections
*b* = 8.0214 (3) Å	θ = 2.9–27.5°
*c* = 14.4832 (7) Å	µ = 0.22 mm^−^^1^
α = 89.864 (3)°	*T* = 120 K
β = 79.530 (2)°	Slab, colourless
γ = 73.812 (3)°	0.27 × 0.13 × 0.03 mm
*V* = 826.78 (7) Å^3^	

### Data collection

**Table:**

Bruker–Nonius CCD camera on κ-goniostat diffractometer	3783 independent reflections
Radiation source: Bruker–Nonius FR591 Rotating Anode	2808 reflections with *I* > 2σ(*I*)
graphite	*R*_int_ = 0.055
Detector resolution: 9.091 pixels mm^-1^	θ_max_ = 27.5°, θ_min_ = 3.0°
φ and ω scans to fill the asymmetric unit	*h* = −9→9
Absorption correction: multi-scan (*SADABS*; Sheldrick, 2007)	*k* = −10→10
*T*_min_ = 0.942, *T*_max_ = 0.993	*l* = −18→18
13798 measured reflections	

### Refinement

**Table:**

Refinement on *F*^2^	Primary atom site location: structure-invariant direct methods
Least-squares matrix: full	Secondary atom site location: difference Fourier map
*R*[*F*^2^ > 2σ(*F*^2^)] = 0.048	Hydrogen site location: inferred from neighbouring sites
*wR*(*F*^2^) = 0.119	H-atom parameters constrained
*S* = 1.05	*w* = 1/[σ^2^(*F*_o_^2^) + (0.0504*P*)^2^ + 0.2877*P*] where *P* = (*F*_o_^2^ + 2*F*_c_^2^)/3
3783 reflections	(Δ/σ)_max_ < 0.001
230 parameters	Δρ_max_ = 0.27 e Å^−^^3^
0 restraints	Δρ_min_ = −0.45 e Å^−^^3^

### Special details

**Table:**

Experimental. *SADABS* was used to perform the Absorption correction Estimated minimum and maximum transmission: 0.6504 0.7456 The given Tmin and Tmax were generated using the *SHELX* SIZE command
Geometry. All e.s.d.'s (except the e.s.d. in the dihedral angle between two l.s. planes) are estimated using the full covariance matrix. The cell e.s.d.'s are taken into account individually in the estimation of e.s.d.'s in distances, angles and torsion angles; correlations between e.s.d.'s in cell parameters are only used when they are defined by crystal symmetry. An approximate (isotropic) treatment of cell e.s.d.'s is used for estimating e.s.d.'s involving l.s. planes.
Refinement. Refinement of *F*^2^ against ALL reflections. The weighted *R*-factor *wR* and goodness of fit *S* are based on *F*^2^, conventional *R*-factors *R* are based on *F*, with *F* set to zero for negative *F*^2^. The threshold expression of *F*^2^ > σ(*F*^2^) is used only for calculating *R*-factors(gt) *etc*. and is not relevant to the choice of reflections for refinement. *R*-factors based on *F*^2^ are statistically about twice as large as those based on *F*, and *R*- factors based on ALL data will be even larger.

### Fractional atomic coordinates and isotropic or equivalent isotropic displacement parameters (Å^2^)

**Table:**

	*x*	*y*	*z*	*U*_iso_*/*U*_eq_	
S1	0.31447 (7)	0.72556 (6)	0.43102 (3)	0.01788 (15)	
O1	0.3403 (2)	0.73723 (17)	0.52605 (9)	0.0238 (3)	
O2	0.1712 (2)	0.65260 (17)	0.41180 (10)	0.0212 (3)	
O3	0.6047 (2)	0.46253 (18)	0.12076 (10)	0.0272 (4)	
O4	0.4283 (2)	0.78465 (18)	0.13671 (9)	0.0239 (3)	
H4	0.4834	0.6873	0.1088	0.036*	
N1	0.5143 (2)	0.6143 (2)	0.36582 (11)	0.0176 (4)	
N2	0.6598 (2)	0.3138 (2)	0.25259 (12)	0.0194 (4)	
H2	0.6433	0.3259	0.3142	0.023*	
C1	0.2807 (3)	0.9323 (2)	0.38337 (14)	0.0171 (4)	
C2	0.1944 (3)	1.0828 (2)	0.43980 (15)	0.0201 (4)	
H2A	0.1576	1.0773	0.5058	0.024*	
C3	0.1629 (3)	1.2423 (2)	0.39801 (15)	0.0215 (5)	
H3	0.1029	1.3468	0.4354	0.026*	
C4	0.2191 (3)	1.2483 (3)	0.30167 (15)	0.0222 (5)	
H4A	0.1977	1.3576	0.2737	0.027*	
C5	0.3058 (3)	1.0979 (3)	0.24565 (14)	0.0202 (4)	
H5	0.3424	1.1045	0.1797	0.024*	
C6	0.3397 (3)	0.9359 (2)	0.28595 (14)	0.0177 (4)	
C7	0.4321 (3)	0.7733 (2)	0.22839 (14)	0.0181 (4)	
C8	0.5104 (3)	0.6205 (2)	0.26674 (13)	0.0176 (4)	
C9	0.5948 (3)	0.4596 (3)	0.20776 (14)	0.0193 (4)	
C10	0.7520 (3)	0.1427 (2)	0.21235 (14)	0.0180 (4)	
C11	0.7367 (3)	0.0876 (3)	0.12370 (15)	0.0212 (4)	
H11	0.6628	0.1666	0.0871	0.025*	
C12	0.8283 (3)	−0.0820 (3)	0.08799 (14)	0.0201 (4)	
C13	0.9391 (3)	−0.1984 (2)	0.14171 (14)	0.0190 (4)	
C14	0.9516 (3)	−0.1413 (2)	0.23036 (14)	0.0198 (4)	
H14	1.0254	−0.2197	0.2673	0.024*	
C15	0.8592 (3)	0.0269 (2)	0.26607 (14)	0.0191 (4)	
H15	0.8692	0.0628	0.3269	0.023*	
C16	0.6865 (3)	0.6405 (3)	0.39200 (16)	0.0245 (5)	
H16A	0.7975	0.5564	0.3557	0.037*	
H16B	0.6829	0.6238	0.4593	0.037*	
H16C	0.6924	0.7587	0.3782	0.037*	
C17	0.8062 (3)	−0.1375 (3)	−0.00753 (15)	0.0282 (5)	
H17A	0.7218	−0.0405	−0.0334	0.042*	
H17B	0.7532	−0.2363	−0.0018	0.042*	
H17C	0.9292	−0.1716	−0.0495	0.042*	
C18	1.0438 (3)	−0.3820 (3)	0.10461 (16)	0.0273 (5)	
H18A	1.1256	−0.4384	0.1478	0.041*	
H18B	1.1202	−0.3793	0.0425	0.041*	
H18C	0.9536	−0.4472	0.0995	0.041*	

### Atomic displacement parameters (Å^2^)

**Table:**

	*U*^11^	*U*^22^	*U*^33^	*U*^12^	*U*^13^	*U*^23^
S1	0.0253 (3)	0.0134 (2)	0.0139 (3)	−0.0045 (2)	−0.0023 (2)	0.00043 (18)
O1	0.0374 (9)	0.0178 (7)	0.0141 (8)	−0.0048 (7)	−0.0048 (7)	−0.0002 (6)
O2	0.0253 (8)	0.0165 (7)	0.0218 (8)	−0.0081 (6)	−0.0015 (6)	0.0003 (6)
O3	0.0358 (9)	0.0250 (8)	0.0154 (8)	−0.0022 (7)	−0.0013 (7)	−0.0013 (6)
O4	0.0338 (9)	0.0206 (7)	0.0137 (8)	−0.0032 (7)	−0.0027 (7)	0.0004 (6)
N1	0.0201 (9)	0.0172 (8)	0.0142 (9)	−0.0028 (7)	−0.0038 (7)	−0.0003 (7)
N2	0.0233 (10)	0.0179 (8)	0.0139 (9)	−0.0021 (7)	−0.0012 (7)	−0.0036 (7)
C1	0.0180 (10)	0.0165 (9)	0.0182 (11)	−0.0057 (8)	−0.0057 (8)	0.0011 (8)
C2	0.0251 (11)	0.0179 (9)	0.0182 (11)	−0.0074 (9)	−0.0038 (9)	−0.0006 (8)
C3	0.0246 (11)	0.0146 (9)	0.0254 (12)	−0.0050 (9)	−0.0060 (9)	−0.0013 (8)
C4	0.0267 (12)	0.0158 (10)	0.0267 (12)	−0.0078 (9)	−0.0088 (9)	0.0056 (8)
C5	0.0236 (11)	0.0224 (10)	0.0173 (11)	−0.0103 (9)	−0.0049 (9)	0.0048 (8)
C6	0.0179 (10)	0.0181 (10)	0.0184 (11)	−0.0066 (8)	−0.0045 (8)	0.0013 (8)
C7	0.0199 (11)	0.0203 (10)	0.0151 (10)	−0.0082 (9)	−0.0021 (8)	0.0014 (8)
C8	0.0204 (11)	0.0188 (10)	0.0131 (10)	−0.0057 (8)	−0.0020 (8)	0.0003 (8)
C9	0.0186 (11)	0.0220 (10)	0.0155 (11)	−0.0046 (9)	−0.0007 (8)	−0.0005 (8)
C10	0.0165 (10)	0.0183 (10)	0.0178 (11)	−0.0050 (8)	0.0000 (8)	−0.0022 (8)
C11	0.0225 (11)	0.0194 (10)	0.0209 (11)	−0.0033 (9)	−0.0066 (9)	0.0010 (8)
C12	0.0222 (11)	0.0223 (10)	0.0164 (11)	−0.0085 (9)	−0.0017 (9)	−0.0026 (8)
C13	0.0204 (11)	0.0172 (9)	0.0187 (11)	−0.0056 (8)	−0.0013 (9)	−0.0010 (8)
C14	0.0204 (11)	0.0194 (10)	0.0204 (11)	−0.0054 (9)	−0.0063 (9)	0.0019 (8)
C15	0.0215 (11)	0.0227 (10)	0.0143 (10)	−0.0084 (9)	−0.0033 (8)	−0.0013 (8)
C16	0.0247 (12)	0.0264 (11)	0.0249 (12)	−0.0087 (9)	−0.0090 (9)	0.0017 (9)
C17	0.0380 (14)	0.0252 (11)	0.0190 (11)	−0.0041 (10)	−0.0071 (10)	−0.0043 (9)
C18	0.0330 (13)	0.0203 (10)	0.0265 (12)	−0.0032 (10)	−0.0071 (10)	−0.0033 (9)

### Geometric parameters (Å, °)

**Table:**

S1—O1	1.4317 (14)	C7—C8	1.368 (3)
S1—O2	1.4326 (14)	C8—C9	1.467 (3)
S1—N1	1.6427 (17)	C10—C15	1.389 (3)
S1—C1	1.7646 (19)	C10—C11	1.394 (3)
O3—C9	1.249 (2)	C11—C12	1.395 (3)
O4—C7	1.336 (2)	C11—H11	0.9500
O4—H4	0.8400	C12—C13	1.405 (3)
N1—C8	1.441 (2)	C12—C17	1.506 (3)
N1—C16	1.485 (3)	C13—C14	1.392 (3)
N2—C9	1.350 (3)	C13—C18	1.511 (3)
N2—C10	1.425 (2)	C14—C15	1.387 (3)
N2—H2	0.8800	C14—H14	0.9500
C1—C2	1.387 (3)	C15—H15	0.9500
C1—C6	1.403 (3)	C16—H16A	0.9800
C2—C3	1.392 (3)	C16—H16B	0.9800
C2—H2A	0.9500	C16—H16C	0.9800
C3—C4	1.388 (3)	C17—H17A	0.9800
C3—H3	0.9500	C17—H17B	0.9800
C4—C5	1.383 (3)	C17—H17C	0.9800
C4—H4A	0.9500	C18—H18A	0.9800
C5—C6	1.400 (3)	C18—H18B	0.9800
C5—H5	0.9500	C18—H18C	0.9800
C6—C7	1.473 (3)		
			
O1—S1—O2	118.91 (8)	O3—C9—C8	120.03 (18)
O1—S1—N1	108.79 (9)	N2—C9—C8	116.48 (17)
O2—S1—N1	107.59 (8)	C15—C10—C11	119.54 (18)
O1—S1—C1	109.51 (9)	C15—C10—N2	117.23 (17)
O2—S1—C1	108.75 (9)	C11—C10—N2	123.23 (18)
N1—S1—C1	101.94 (9)	C10—C11—C12	120.92 (19)
C7—O4—H4	109.5	C10—C11—H11	119.5
C8—N1—C16	114.78 (16)	C12—C11—H11	119.5
C8—N1—S1	112.57 (13)	C11—C12—C13	119.55 (18)
C16—N1—S1	115.69 (13)	C11—C12—C17	119.35 (18)
C9—N2—C10	127.93 (17)	C13—C12—C17	121.10 (18)
C9—N2—H2	116.0	C14—C13—C12	118.72 (18)
C10—N2—H2	116.0	C14—C13—C18	120.17 (18)
C2—C1—C6	122.11 (18)	C12—C13—C18	121.12 (18)
C2—C1—S1	121.06 (15)	C15—C14—C13	121.66 (19)
C6—C1—S1	116.80 (14)	C15—C14—H14	119.2
C1—C2—C3	118.78 (19)	C13—C14—H14	119.2
C1—C2—H2A	120.6	C14—C15—C10	119.61 (18)
C3—C2—H2A	120.6	C14—C15—H15	120.2
C4—C3—C2	119.89 (18)	C10—C15—H15	120.2
C4—C3—H3	120.1	N1—C16—H16A	109.5
C2—C3—H3	120.1	N1—C16—H16B	109.5
C5—C4—C3	121.17 (18)	H16A—C16—H16B	109.5
C5—C4—H4A	119.4	N1—C16—H16C	109.5
C3—C4—H4A	119.4	H16A—C16—H16C	109.5
C4—C5—C6	120.08 (19)	H16B—C16—H16C	109.5
C4—C5—H5	120.0	C12—C17—H17A	109.5
C6—C5—H5	120.0	C12—C17—H17B	109.5
C5—C6—C1	117.96 (18)	H17A—C17—H17B	109.5
C5—C6—C7	121.49 (18)	C12—C17—H17C	109.5
C1—C6—C7	120.55 (17)	H17A—C17—H17C	109.5
O4—C7—C8	122.55 (18)	H17B—C17—H17C	109.5
O4—C7—C6	115.15 (17)	C13—C18—H18A	109.5
C8—C7—C6	122.27 (18)	C13—C18—H18B	109.5
C7—C8—N1	120.84 (17)	H18A—C18—H18B	109.5
C7—C8—C9	120.78 (18)	C13—C18—H18C	109.5
N1—C8—C9	118.37 (16)	H18A—C18—H18C	109.5
O3—C9—N2	123.49 (18)	H18B—C18—H18C	109.5
			
O1—S1—N1—C8	−170.25 (12)	C6—C7—C8—N1	−3.3 (3)
O2—S1—N1—C8	59.69 (14)	O4—C7—C8—C9	−0.2 (3)
C1—S1—N1—C8	−54.62 (14)	C6—C7—C8—C9	177.62 (17)
O1—S1—N1—C16	−35.53 (16)	C16—N1—C8—C7	−91.7 (2)
O2—S1—N1—C16	−165.58 (13)	S1—N1—C8—C7	43.5 (2)
C1—S1—N1—C16	80.10 (15)	C16—N1—C8—C9	87.5 (2)
O1—S1—C1—C2	−31.1 (2)	S1—N1—C8—C9	−137.36 (16)
O2—S1—C1—C2	100.31 (18)	C10—N2—C9—O3	1.4 (3)
N1—S1—C1—C2	−146.23 (17)	C10—N2—C9—C8	−178.11 (17)
O1—S1—C1—C6	150.83 (15)	C7—C8—C9—O3	3.8 (3)
O2—S1—C1—C6	−77.72 (17)	N1—C8—C9—O3	−175.38 (18)
N1—S1—C1—C6	35.73 (17)	C7—C8—C9—N2	−176.68 (18)
C6—C1—C2—C3	1.0 (3)	N1—C8—C9—N2	4.2 (3)
S1—C1—C2—C3	−176.88 (15)	C9—N2—C10—C15	159.47 (19)
C1—C2—C3—C4	−0.6 (3)	C9—N2—C10—C11	−21.5 (3)
C2—C3—C4—C5	0.3 (3)	C15—C10—C11—C12	−0.3 (3)
C3—C4—C5—C6	−0.5 (3)	N2—C10—C11—C12	−179.32 (18)
C4—C5—C6—C1	0.9 (3)	C10—C11—C12—C13	−0.7 (3)
C4—C5—C6—C7	−179.85 (18)	C10—C11—C12—C17	179.17 (19)
C2—C1—C6—C5	−1.2 (3)	C11—C12—C13—C14	1.1 (3)
S1—C1—C6—C5	176.80 (15)	C17—C12—C13—C14	−178.73 (19)
C2—C1—C6—C7	179.56 (19)	C11—C12—C13—C18	−178.68 (19)
S1—C1—C6—C7	−2.4 (3)	C17—C12—C13—C18	1.5 (3)
C5—C6—C7—O4	−19.6 (3)	C12—C13—C14—C15	−0.6 (3)
C1—C6—C7—O4	159.62 (18)	C18—C13—C14—C15	179.19 (19)
C5—C6—C7—C8	162.50 (19)	C13—C14—C15—C10	−0.4 (3)
C1—C6—C7—C8	−18.3 (3)	C11—C10—C15—C14	0.8 (3)
O4—C7—C8—N1	178.98 (17)	N2—C10—C15—C14	179.89 (17)

### Hydrogen-bond geometry (Å, °)

**Table:**

*D*—H···*A*	*D*—H	H···*A*	*D*···*A*	*D*—H···*A*
O4—H4···O3	0.84	1.80	2.545 (2)	146
N2—H2···O1^i^	0.88	2.39	3.231 (2)	161

Symmetry codes: (i) −*x*+1, −*y*+1, −*z*+1.
